# Cadmium-Induced Apoptosis in Primary Rat Cerebral Cortical Neurons Culture Is Mediated by a Calcium Signaling Pathway

**DOI:** 10.1371/journal.pone.0064330

**Published:** 2013-05-31

**Authors:** Yan Yuan, Chen-yang Jiang, Hui Xu, Ya Sun, Fei-fei Hu, Jian-chun Bian, Xue-zhong Liu, Jian-hong Gu, Zong-ping Liu

**Affiliations:** College of Veterinary Medicine, Yang Zhou University, Yangzhou, China; Hertie Institute for Clinical Brain Research and German Center for Neurodegenerative Diseases, Germany

## Abstract

Cadmium (Cd) is an extremely toxic metal, capable of severely damaging several organs, including the brain. Studies have shown that Cd disrupts intracellular free calcium ([Ca^2+^]_i_) homeostasis, leading to apoptosis in a variety of cells including primary murine neurons. Calcium is a ubiquitous intracellular ion which acts as a signaling mediator in numerous cellular processes including cell proliferation, differentiation, and survival/death. However, little is known about the role of calcium signaling in Cd-induced apoptosis in neuronal cells. Thus we investigated the role of calcium signaling in Cd-induced apoptosis in primary rat cerebral cortical neurons. Consistent with known toxic properties of Cd, exposure of cerebral cortical neurons to Cd caused morphological changes indicative of apoptosis and cell death. It also induced elevation of [Ca^2+^]_i_ and inhibition of Na^+^/K^+^-ATPase and Ca^2+^/Mg^2+^-ATPase activities. This Cd-induced elevation of [Ca^2+^]_i_ was suppressed by an IP_3_R inhibitor, 2-APB, suggesting that ER-regulated Ca^2+^ is involved. In addition, we observed elevation of reactive oxygen species (ROS) levels, dysfunction of cytochrome oxidase subunits (COX-I/II/III), depletion of mitochondrial membrane potential (ΔΨm), and cleavage of caspase-9, caspase-3 and poly (ADP-ribose) polymerase (PARP) during Cd exposure. Z-VAD-fmk, a pan caspase inhibitor, partially prevented Cd-induced apoptosis and cell death. Interestingly, apoptosis, cell death and these cellular events induced by Cd were blocked by BAPTA-AM, a specific intracellular Ca^2+^ chelator. Furthermore, western blot analysis revealed an up-regulated expression of Bcl-2 and down-regulated expression of Bax. However, these were not blocked by BAPTA-AM. Thus Cd toxicity is in part due to its disruption of intracellular Ca^2+^ homeostasis, by compromising ATPases activities and ER-regulated Ca^2+^, and this elevation in Ca^2+^ triggers the activation of the Ca^2+^-mitochondria apoptotic signaling pathway. This study clarifies the signaling events underlying Cd neurotoxicity, and suggests that regulation of Cd-disrupted [Ca^2+^]_i_ homeostasis may be a new strategy for prevention of Cd-induced neurodegenerative diseases.

## Introduction

Cadmium (Cd) is an extremely toxic metal commonly found in industrial workplaces. It is also a food contaminant and a major component of cigarette smoke. It is toxic even at low doses since the metal accumulates and has a long biological half-life in humans (10–30 years) [1]. Cd is toxic to many organs, including liver, kidney, lung, testis and brain [2]–[7]. In addition, it can enter the brain parenchyma and neurons causing neurological alterations in humans and animal models, leading to lower attention, hypernociception, olfactory dysfunction and memory deficits [8], [9]. Increasing evidence has demonstrated that Cd is a possible etiological factor of neurodegenerative diseases, such as Alzheimer’s disease (AD) and Parkinson’s disease (PD) [10], [11].

Studies have shown that Cd disrupts calcium homeostasis, leading to apoptosis in a variety of cells [12]–[16]. Recently, Xu et al [17] have demonstrated that Cd-induced apoptosis in primary murine neurons occurs through a calcium-dependent pathway. Calcium is a universal messenger regulating many physiological and pathological functions, such as secretion, contraction, metabolism, gene transcription, and cell death [18], [19]. The cellular uptake of Cd occurs mainly through the Ca^2+^ channels (including both voltage-gated and receptor-operated Ca^2+^ channels) and Cd is a potent Ca^2+^ channel blocker and inhibits Ca^2+^ cellular uptake [20], [21]. The cellular toxicity of Cd is, in part, related to the alteration of intracellular calcium homeostasis, which can competitively reduce extracellular calcium influx or increase intracellular calcium concentration ([Ca^2+^]_i_) by inhibiting calcium-dependent ATPase or by stimulating the inositol triphosphate pathway [13], [22], [23]. Moreover, a number of studies have demonstrated that Cd interacts with the functions of many Ca^2+^-dependent enzymes such as endonuclease and regulatory proteins such as protein kinase C (PKC), mitogen-activated protein kinase, and phospholipase C, thus interfering with calcium homeostasis [21], [24]–[26]. Intracellular calcium homeostasis is very important in maintaining the normal function of the cell, in that variations in the concentration of calcium in cells can determine cell survival or death. For example, a high [Ca^2+^]_i_ can cause disruption of mitochondrial Ca^2+^ equilibrium, which results in reactive oxygen species (ROS) formation due to the stimulation of electron flux along the electron transport chain (ETC) [27]. Under oxidative stress, mitochondrial Ca^2+^ accumulation can switch from a physiologically beneficial process to a cell death signal [28].

Cd can also induce apoptosis *in* *vitro* through direct targeting of mitochondria [29]. Recent studies have revealed the ability of Cd to compromise the mitochondrial membrane potential (ΔΨm) [16], [30]. ΔΨm triggers the release of proteins that are normally confined to the mitochondrial intermembrane space (IMS) into the cytosol. The proteins released include cytochrome c (which stimulates the cytosolic assembly of the apoptosome, the caspase-9 activation complex) and AIF (apoptosis-inducing factor) [31]. Finally, the activation of catabolic hydrolases, mainly caspases and nucleases, causes the cleavage of important cellular targets and leads to apoptotic cell death.

Moreover, it has been proven that Cd-induced neurotoxicity may be due to excessive ROS production in cerebral cortical neurons [4] and in various types of cells [17], [32], [33]. In addition, Cd-induced apoptosis of neuronal cells is triggered by elevated [Ca^2+^]_i_, leading to ROS induction and subsequent activation of caspase signaling pathway [17].

Thus Cd toxicity appears to involve Ca^2+^ signaling, mitochondrial-mediated apoptosis, and ROS production. However, it remains to be clarified how these events are inter-related and what the details are regarding the role of the mitochondrial in Cd toxicity. Therefore, in this study, we investigated the role of Ca^2+^ signaling pathway and downstream events in Cd-induced apoptosis in primary rat cerebral cortical neurons.

## Materials and Methods

### Reagents

Fetal calf serum (FCS) was obtained from Hyclone Laboratories (Logan, UT, USA). NEUROBASAL™ Medium, B27 Supplement, Trizol reagent, RNAase inhibitor, dNTPs, reverse primer and SuperScript™ III reverse transcriptase were purchased from Invitrogen (Grand Island, NY, USA). Dulbecco’s modified Eagle’s medium (DMEM)-F_12_ (1∶1), Fluo-4/AM, Rhodamine 123 (Rh123), cadmium acetate (CdAc_2_), 3-(4,5-dimethylthiazol-2-yl)-2,5-diphenyl tetrazolium bromide (MTT), 2-Aminoethoxydiphenyl borate (2-APB), 2′,7′-dichloro-dihydrofluorescein diacetate (H_2_-DCF-DA), antibody for β-actin, Hoechst 33258 staining, trypsin, penicillin/streptomycin were purchased from Sigma Chemical Co. (St. Louis, MO, USA). Antibodies against cleaved caspase-9, cleaved caspase-3, cleaved poly (ADP-ribose) polymerase (PARP), Bcl-2 and Bax were obtained from Cell Signaling Technology (Boston, MA, USA). N-benzyloxycarbonyl-Val-Ala-Asp-fluoromethylketone (Z-VAD-fmk) was supplied by Biovision (Mountain View, CA). The 1, 2-bis (2-amino-phenoxy) ethane-N,N,N′,N′-tetraacetic acid-tetraacetoxymethyl ester (BAPTA-AM) was from Alexis Biochemicals Corporation (San Diego, CA). Horseradish peroxidase (HRP)-conjugated goat anti-rabbit immunoglobulin G (IgG) was from Santa Cruz Biotechnology (Santa Cruz, CA, USA). Na^+^/K^+^-APTase, and Ca^2+^/Mg^2+^-APTase analysis kits were obtained from Jiancheng Bioengineering Institute (Nanjing, China). Enhanced chemiluminescence solution was from Thermo Fisher Scientific (Waltham, MA). All other reagents were of analytical grade.

### Cell Isolation and Culture

Fetal Sprague-Dawley rats of 18–19 days of gestation were obtained from Laboratory Animal Center in Yangzhou University (Yangzhou, China). This study was carried out in strict accordance with the recommendations in the Guide for the Care and Use of Laboratory Animals of the National Research Council. The animal care and use committee of Yangzhou University approved all experiments and procedures conducted on the animals (approval ID: SYXK (Su) 2007–0005). Primary rat cerebral cortical neurons were cultured from fetal Sprague-Dawley rats of 18–19 days of gestation, as described [34]. Isolated cells were seeded at a density of 1×10^6^ cells/well in 6-well plates or 2×10^4^ cells/well in 96-well plates coated with 100 mg/L poly-L-lysine in NEUROBASAL™ Medium supplemented with 2% B27 Supplement, 1 mM L-glutamine, 100 U/ml penicillin and 100 U/ml streptomycin. These cells were grown in a humid incubator (37°C, 5% CO_2_), with the media replaced every 3 days. The cells were used for experiments after 6 days of culture.

### Cell Viability Assay and Morphology

Cells were seeded at a density of 2×10^4^ cells/well in 96-well plates. Cells were treated with 0–25 µM Cd for 12 and 24 h, or with/without 10 µM Cd for 12 and 24 h following pre-incubation with/without BAPTA-AM (10 µM) for 30 min or Z-VAD-fmk (100 µM) for 1 h with 6 replicates of each treatment. At the designated time points, cell viability was measured by the MTT assay, which is based on the conversion of the tetrazolium salt to the colored product, formazan. In brief, 20 µl MTT solution (5.0 g/L in PBS) was added into each well of the 96-well plates (containing 100 µl medium and cells) 4 h before the end of incubation. The supernatant was then discarded, and 150 µl DMSO was added to dissolve the formazan. The absorbance was measured at 570/630 nm by the microplate reader (Sunrise, Austria).

For cell morphological analysis, cells were seeded at a density of 1×10^6^ cells/well in six-well plates. At day 6, various Cd doses (0, 5, 10 and 20 µM) or Cd (10 µM) following 30 min of BAPTA-AM (10 µM) or 1 h of Z-VAD-fmk (100 µM) pre-incubation was added. After incubation for 24 h, the culture plates were examined and photographed by a DMI300 inverted phase microscopy (Leica, Germany) (400×) equipped with the Quick Imaging system.

### Hoechst 33258 Staining

Apoptotic morphological changes in the nuclear chromatin were examined using Hoechst 33258 staining. The cells were fixed with 4% paraformaldehyde, stained with Hoechst 33258 (5 mg/L) for 10 min and examined by fluorescence microscopy to analyze cell chromatin condensation.

### Analysis of Intracellular Free Ca^2+^concentration ([Ca^2+^]_i_)

Cells were cultured in six-well plates and pretreated with BAPTA-AM (10 µM), or 2-APB (50 µM) for 30 min, followed by treatment with various Cd concentrations (0, 5, 10 and 20 µM) for another 12 h. Fluo-4/AM was used as an intracellular free Ca^2+^ fluorescent probe to analyze [Ca^2+^]_i_ in Cd-exposed cerebral cortical neurons. In short, the harvested cells were incubated with Fluo-4/AM (5 µmol/L final concentration) for 30 min at 37°C in the dark, washed with PBS, and analyzed on a BD-FACS Aria flow cytometry. Intracellular [Ca^2+^]_i_ levels were represented by fluorescent intensity. Fluorescent intensity was recorded by excitation at 494 nm and emission at 516 nm. The data were analyzed by Cell Quest program (Becton Dickinson), and the mean fluorescence intensity was obtained by histogram statistics.

### Measurement of Mitochondrial Membrane Potential (ΔΨm) and Reactive Oxygen Species (ROS) Production

Cells were cultured in six-well plates and pretreated with BAPTA-AM (10 µM) for 30 min, followed by treatment with various Cd concentrations (0, 5, 10 and 20 µM) for another 12 h. For the detection of mitochondrial ΔΨ, the harvested cells were incubated with Rh123 (10 mg/L final concentration) for 30 min in the dark at 37°C, harvested and re-suspended in PBS and analyzed on a BD-FACS Aria flow cytometry. The mitochondrial ΔΨ was based on Rh123 fluorescence intensity which was recorded by excitation at 488∼505 nm and emission at 530 nm. Generation of ROS was monitored by using H_2_-DCF-DA, a redox-sensitive fluorescent dye. Briefly, the harvested cells were incubated with H_2_-DCF-DA (50 µmol/L final concentration) for 30 min in the dark at 37°C. After treatment, cells were immediately washed twice, re-suspended in PBS, and analyzed on a BD-FACS Aria flow cytometry. ROS generation was based on fluorescent intensity which was recorded by excitation at 504 nm and emission at 529 nm. Each measurement was conducted on 10,000 events and analyzed on Cell Quest software (Becton Dickinson), and the mean fluorescence intensity was obtained by histogram statistics.

### Activities of Na^+^/K^+^-ATPase, and Ca^2+^/Mg^2+^-ATPase

The harvested cells were homogenized in ice-cold physiological saline in an ultrasonic disintegrator. The cell homogenates were centrifuged at 1,000 g for 10 min, and supernatants were obtained. The concentration of protein in the supernatant was determined by the Folin phenol method, using bovine serum albumin as a standard. The ATPases activities were assayed by the quantization of phosphonium ions, which were performed in accordance with the ATPase detection protocol.

### RNA Extraction and Real-time Quantitative PCR (qRT-PCR)

Total RNA was isolated from cerebral cortical neurons using Trizol reagent protocol (Invitrogen). A total of 5 µg of RNA was reverse transcribed into cDNA and amplified by qPCR using an ABI 7500 Real Time PCR System (Applied Biosystem, USA). Primer pairs specific for cytochrome oxidase subunits (COX-I/II/III) genes in rat, and β-actin were designed as shown in Table 1 and synthesized by Invitrogen (Shanghai, China). The calculations of the relative expression of COX-I, COX-II, and COX-III in the (normalized) experimental groups versus the (normalized) control group were compared using confidence intervals for ratios calculated by the comparative C_T_ method (threshold cycle number at the cross-point between amplification plot and threshold) [35] and values were normalized to an internal β-actin control.

**Table 1:** Primers used for qRT-PCR.

Gene Name	Primer (5'–3')	Product (base pairs)	Accession No.
COX-I	F: AGCTGGCTTCGTCCACTGAT	223 bp	NM_AY172581
	R: GGCCGTAAGTGAGATGAATG		
COX-II	F:AAGACGCCACATCACCTATCAT	151 bp	NM_AY172581
	R: TCTTGGGCGTCTATTGTGCTT		
COX-III	F: CAGGAGCCCTATCAGCTCTTC	159 bp	NM_AY172581
	R: TGTGGTGGCCTTGGTATGTT		
β-actin	F: CTCATGCCATCCTGCGTCT	116 bp	NM_031144
	R: ACGCACGATTTCCCTCTCA		

### Western Blotting Analysis

After treatment, cells were briefly washed twice with cold PBS. Cells were lysed in RIPA buffer (50 mM Tris, pH 7.4; 150 mM NaCl; 1% NP-40; 0.1% SDS) on ice. Lysates were sonicated for 10 sec and centrifuged at 12,000 g for 10 min at 4°C. Protein concentration was determined by bicinchoninic acid assay with bovine serum albumin as standard. Equivalent amounts of protein were separated on 10–15% SDS–polyacrylamide gels and transferred to nitrocellulose membranes. Membranes were incubated with TBS containing 0.05% Tween 20 and 5% nonfat dry milk to block nonspecific binding and were incubated overnight at 4°C with antibodies against cleaved caspase-3, cleaved caspase-9, cleaved PARP, Bcl-2 and Bax (1∶1,000 dilution) or β-actin (1∶2,000 dilution). Detection was performed with the appropriate horseradish peroxidase-conjugated secondary antibodies (1∶5,000 dilution) and enhanced chemiluminescence reagent. All assays were performed in duplicate.

### Statistical Analysis

Values were expressed as the mean ± standard deviation (SD) and significance was calculated by Student’s t-test. A *P*<0.05 was considered statistically significant.

## Results

### Cd Triggers Apoptosis in Cerebral Cortical Neurons

**Figure 1:**
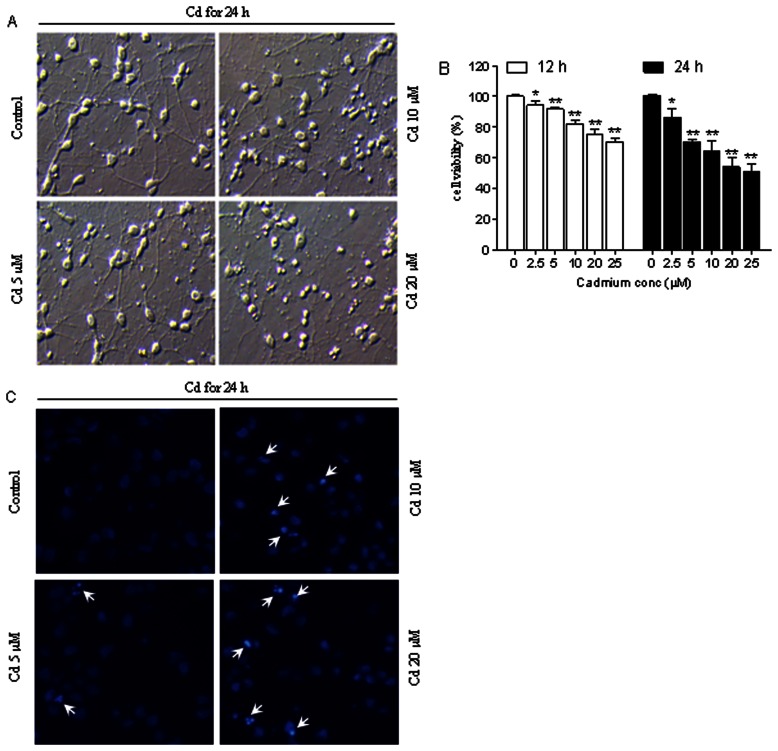
Cd induces apoptosis in cerebral cortical neurons. (A) Morphological alterations in cerebral cortical neurons treated with different concentrations of Cd (0, 5, 10 and 20 µM) for 24 h. Cell morphology was assessed using a LEICA inverted phase-contrast microscope (400×) equipped with Quick Imaging system. All experiments were performed in duplicate. (B) Cell viability of cerebral cortical neurons, treated with 0–25 µM Cd for 12 and 24 h, was evaluated by MTT assay. Results are presented as mean ± SD (n = 6). *Statistical significance between control and Cd treatment (2.5, 5, 10, 20 and 25 µM); *P<0.05, **P<0.01 compared to respective control, using Student’s t-test. (C) Cd induced apoptotic morphological changes in cerebral cortical neurons. Cerebral cortical neurons were incubated with 0–20 µM Cd for 24 h, stained with Hoechst 33258. Cell morphology was analyzed by fluorescence microscopy (arrows, apoptotic cells). The original magnification is 400×. All experiments were performed in duplicate.

We evaluated Cd toxicity on cerebral cortical neurons initially by phase-contrast microscopy. More round or shrunken cells appeared in cerebral cortical neurons, when exposed to increasing concentrations of Cd (5, 10, 20 µM) for 24 h (Fig. 1A). Moreover, cell density and neural network were decreased, with cells showing a great loss of neuronal integrity based on the disappearance of axons and dendrites caused by increasing concentrations of Cd. This is consistent with results from MTT assay which further demonstrate Cd-induced loss of cell viability in cerebral cortical neurons (Fig. 1B).

Apoptotic morphological changes induced by Cd in cerebral cortical neurons were assessed by fluorescence microscopy analysis of Hoechst 33258 staining (Fig.1C). In the control group, the majority of cells had uniformly stained nuclei, and the chromatin of normal nuclei was unaltered and spread uniformly throughout the entire nucleus. After exposure to CdAc_2_ (5, 10 and 20 µmol/L) for 24 h, the cells showed nuclear morphological changes typical of apoptosis, i.e., condensed nuclear chromatin and fragmented nuclei characterized by a scattered, drop-like structure. The nuclei of apoptotic cells were smaller than the nuclei of intact cells.

### Cd Induces Calcium-dependent Apoptosis

**Figure 2:**
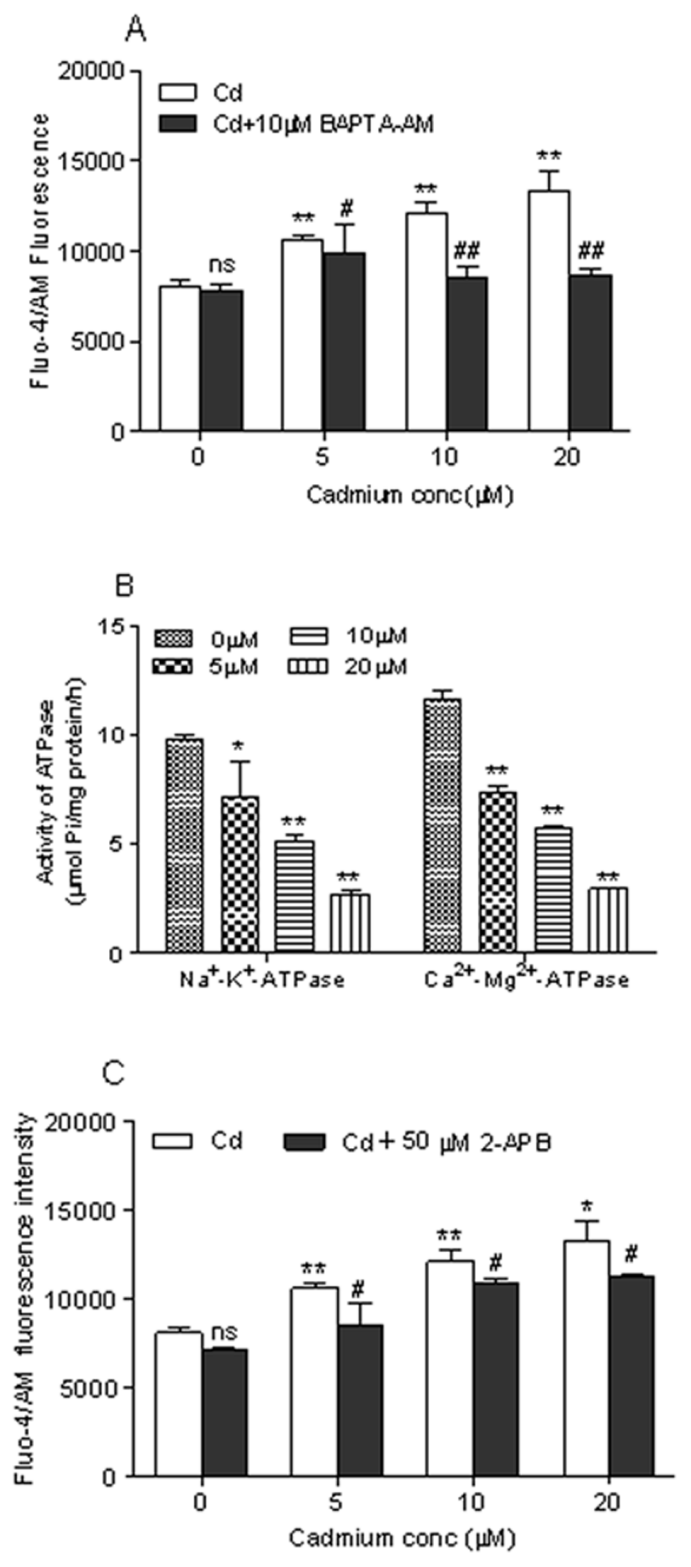
Cd-induced intracellular [Ca^2+^]_i_ elevation in cerebral cortical neurons. (A) Cd-induced elevation of [Ca^2+^]_i_ was diminished by BAPTA-AM. Cells were pretreated with 10 µM BAPTA-AM for 30 min, followed by treatment with Cd for another 12 h and incubated with 5 µM Fluo-4 AM dye for a total of 30 min before analysis by flow cytometry. Results were expressed as mean fluorescence. Each bar represents mean ± SD (n = 6). *Statistical significance between control and Cd treatment (5, 10 and 20 µM); ^#^Statistical significance between cells cultured in the absence and presence of BAPTA-AM. *ns* Not significant; **P<0.01; ^#^ *P*<0.05, ^##^ *P*<0.01, using Student’s t-test. (B) Inhibition of Na^+^/K^+^-ATPase and Ca^2+^/Mg^2+^-ATPase activities by Cd in cerebral cortical neurons. The neurons were treated with Cd (0,5, 10 and 20 µM) for 12 h to measure the assays. Data are mean ± SD of three experiments from cells of different cultures, each one performed in triplicate. *P<0.05, **P<0.01 compared to control, using Student’s t-test. (C) Calcium released from ER through IP_3_R channels is a major cause of Cd-induced elevation of [Ca^2+^]_i_. Cells were pretreated with 50 µM 2-APB for 30 min, followed by treatment with Cd for another 12 h. The cytosolic calcium level was analyzed by Fluo-4 AM staining and flow cytometry. Results are expressed as mean fluorescence. Each bar represents mean ± SD (n = 6). *Statistical significance between control and Cd treatment (5, 10 and 20 µM); ^#^Statistical significance between cells cultured in the absence and presence of 2-APB. *ns* Not significant; *P<0.05, **P<0.01; ^#^ *P*<0.05, using Student’s t-test.

To determine the cellular calcium level after treatment of cerebral cortical neurons with Cd, [Ca^2+^]_i_ was measured by flow cytometry analysis of cells stained with Fluo-4 AM, a calcium indicator dye. We found that treatment with Cd (5, 10, 20 µM) resulted in a concentration-dependent increase of [Ca^2+^]_i_ in cerebral cortical neurons (Fig. 2A). To verify the role of [Ca^2+^]_i_ as a key second messenger, cells were pre-loaded with 10 µM BAPTA-AM for 30 min. BAPTA-AM is an effective membrane-permeable intracellular Ca^2+^ chelator, and becomes trapped in the cells after cytoplasmic hydrolysis. As shown in Fig. 2A, chelating intracellular Ca^2+^ with BAPTA-AM prevented the elevation of [Ca^2+^]_i_, demonstrating that the release of intracellular Ca^2+^ is essential for Cd-induced [Ca^2+^]_i_ overloading. To explore other factors contributing to the calcium overload, we studied the effect of Cd on the activities of ATPases. As shown in Fig. 2B, treatment of cerebral cortical neurons with Cd resulted in a significant loss in the activities of ATPases (*P*<0.05 or *P*<0.01), which occurred in a dose-dependent manner. When exposed to 5, 10 and 20 µM of Cd for 12 h, the Na^+^/K^+^-ATPase activity decreased to 70.1%, 52.5% and 27.2% of the control value while the Ca^2+^/Mg^+^-ATPase activity decreased to 62.6%, 49.0% and 25.5% of the control value, respectively.

The endoplasmic reticulum (ER) is one of the major calcium storage compartments in cells. To examine the role of the ER in Cd-induced elevation of [Ca^2+^]_i_, we incubated neurons with 2-APB, a blocker of the ER calcium channel (inositol-1, 4, 5-trisphosphate receptor, IP_3_R). As shown in Fig. 2C, we observed that the elevation of [Ca^2+^]_i_ induced by Cd was suppressed by 2-APB after treatment with Cd for 12 h. Taken together, these results demonstrated that [Ca^2+^]_i_ elevation induced by Cd in cerebral cortical neurons is linked to the release of calcium from the ER.

**Figure 3:**
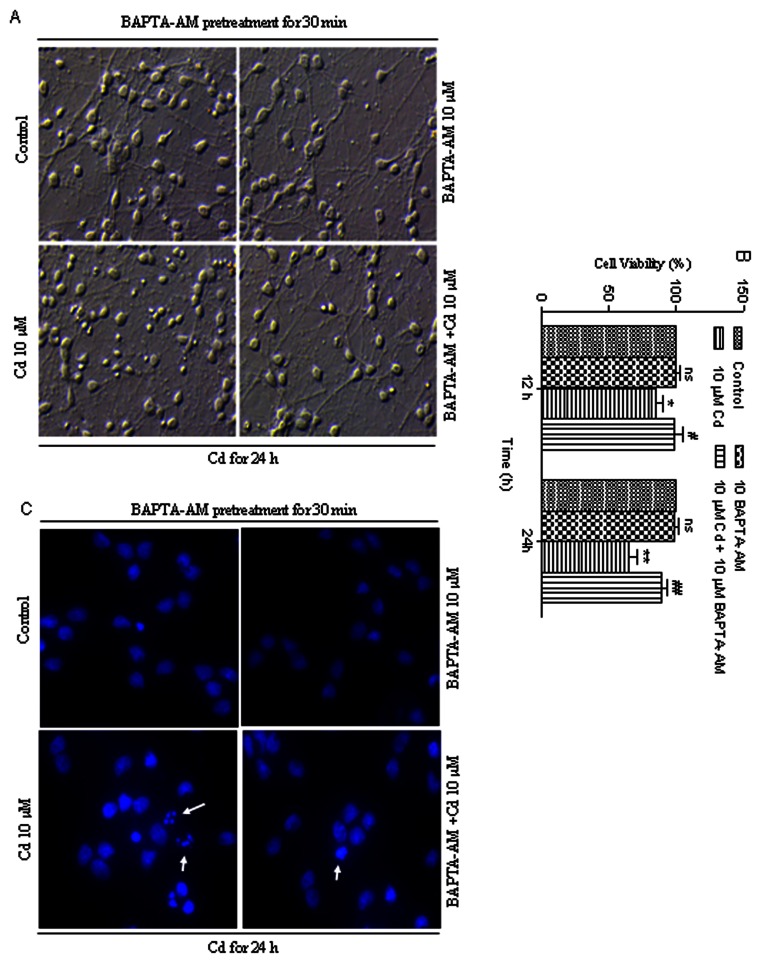
Cd-induced apoptosis arises through calcium-mediated signaling. (A) Morphological alterations in cerebral cortical neurons treated with/without Cd (10 µM) in the presence or absence of BAPTA-AM (10 µM) for 24 h. Morphology of cerebral cortical neurons was assessed using a Leica inverted phase-contrast microscope (400×) equipped with digital camera. All experiments were performed twice. (B) Cell viability of cerebral cortical neurons treated with/without Cd (10 µM) in the presence or absence of BAPTA-AM (10 µM) for 12 and 24 h. Results are presented as mean± SD (n = 6). *Statistical significance between control and Cd treatment (5, 10 and 20 µM); ^#^Statistical significance between cells cultured in the absence and presence of BAPTA-AM. *ns* Not significant; *P<0.05, **P<0.01; ^#^ *P*<0.05, ^##^ *P*<0.01, using Student’s t-test. (C) Cd-induced apoptosis were suppressed by BAPTA-AM. Cells were pretreated with BAPTA-AM (10 µM) for 30 min, followed by treatment with Cd (10 µM) for another 24 h to determine apoptosis by Hoechst 33258 staining under a fluorescence microscope (arrows, apoptotic cells). The original magnification is 400×. All experiments were performed twice.

Next, to further determine the role of calcium in the regulation of Cd-induced apoptosis, cerebral cortical neurons were incubated with/without Cd (10 µM) in the absence or presence of BAPTA-AM (10 µM). As shown in Fig. 3A, Cd alone (10 µM) induced cell rounding and shrinkage, and BAPTA-AM itself did not alter cell shape. However, BAPTA-AM obviously blocked Cd-induced morphological changes. Furthermore, MTT assay results (Fig. 3B) further demonstrated that BAPTA-AM in part can suppress Cd-induced loss of cell viability in Cd-exposed cerebral cortical neurons. These results suggest that Cd-induced neuronal apoptosis might be associated with its induction of [Ca^2+^]_i_ elevation (Fig. 3C).

### Cd-elevated [Ca^2+^]_i_ Induces ROS, Triggering Apoptosis of Cerebral Cortical Neurons

**Figure 4:**
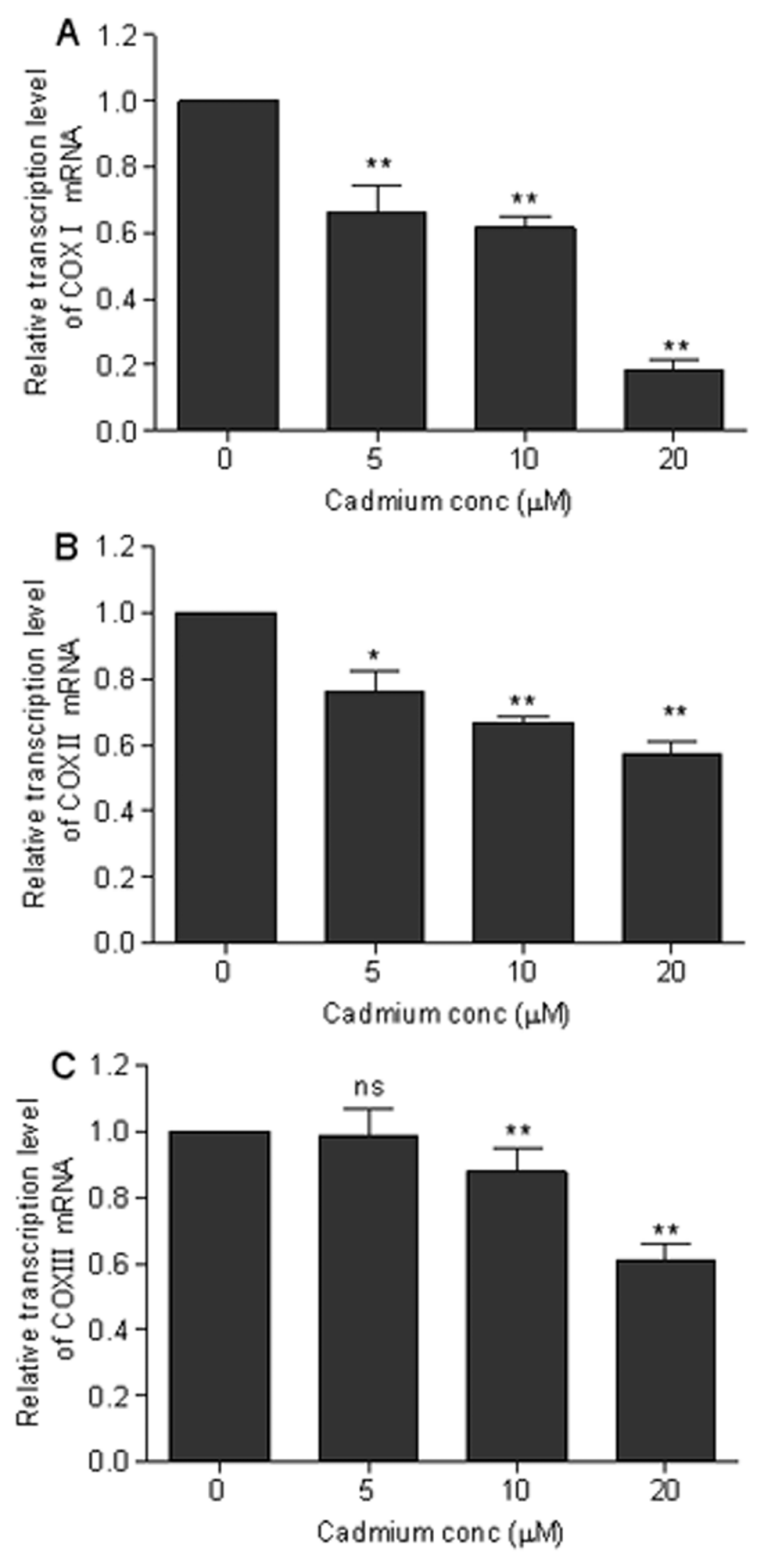
Relative transcription levels of the genes in neurons exposed to Cd for 6 h. The transcription levels were calculated relative to the control group, whose expression level was set to one. (A) Cytochrome oxidase subunit I (COX-I). (B) Cytochrome oxidase subunit II (COX-II). (C) Cytochrome oxidase subunit III (COX-III). Data represent mean ± SD determined by the comparative C_T_ method (n = 6). *ns* Not significant; *P<0.05, **P<0.01; statistical significance between control and Cd treatment (5, 10 and 20 µM ).

**Figure 5:**
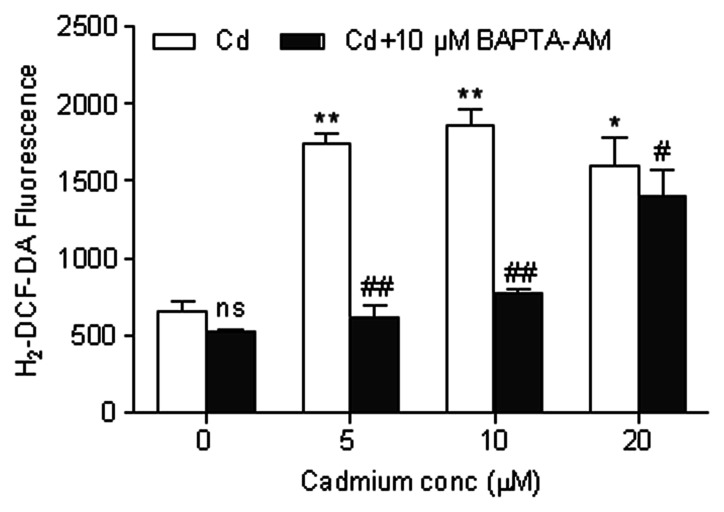
Cd-elevated [Ca^2+^]_i_ induces ROS in cerebral cortical neurons. Cells were pretreated with BAPTA-AM (10 µM) for 30 min, followed by treatment with Cd for another 12 h. The generation of ROS was analyzed by H_2_-DCF-DA staining using flow cytometry. Results were expressed as mean fluorescence. Each bar represents mean ± SD (n = 6). *Statistical significance between control and Cd treatment (5, 10 and 20 µM); ^#^Statistical significance between cells cultured in the absence and presence of BAPTA-AM. *ns* Not significant; *P<0.05, **P<0.01; ^#^ *P*<0.05, ^##^ *P*<0.01 using Student’s t-test.

Studies have shown that Cd-induced neuronal apoptosis is attributed to the induction of ROS [36], [37]. In this study, we examined whether ROS production is involved in the Cd-induced apoptosis in cerebral cortical neurons. The expression levels of COX-I, II, and III are closely related to the generation of ROS [38]. COX is a terminal enzyme in the mitochondrial respiratory chain involved in aerobic ATP production, and has been used in several studies to assess the mitochondrial function and respiratory responses of organisms to toxic chemicals, especially Cd [39]–[41]. Thus we assessed the effects of Cd treatment on the mRNA expression levels of mitochondrial COX- I/II/III. As shown in Fig. 4, the relative expression levels of mitochondrial COX-I/II/III were all significantly (*P*<0.05 or *P*<0.01) decreased in the cerebral cortical neurons after 6 h of Cd exposure. In addition, production of ROS was detected in cells treated with Cd for 12 h (Fig. 5). As expected, BAPTA-AM significantly attenuated Cd-induced production of ROS, which is in agreement with our observation that BAPTA-AM prevented Cd-induced apoptosis and cell death in cerebral cortical neurons (Fig. 3). These data suggest that Cd elevates [Ca^2+^]_i_ level, which induces ROS and in turn trigger apoptosis in cerebral cortical neurons.

### Cd-induced Apoptosis Occurs Through Ca^2+^-mitochondria Signaling

**Figure 6:**
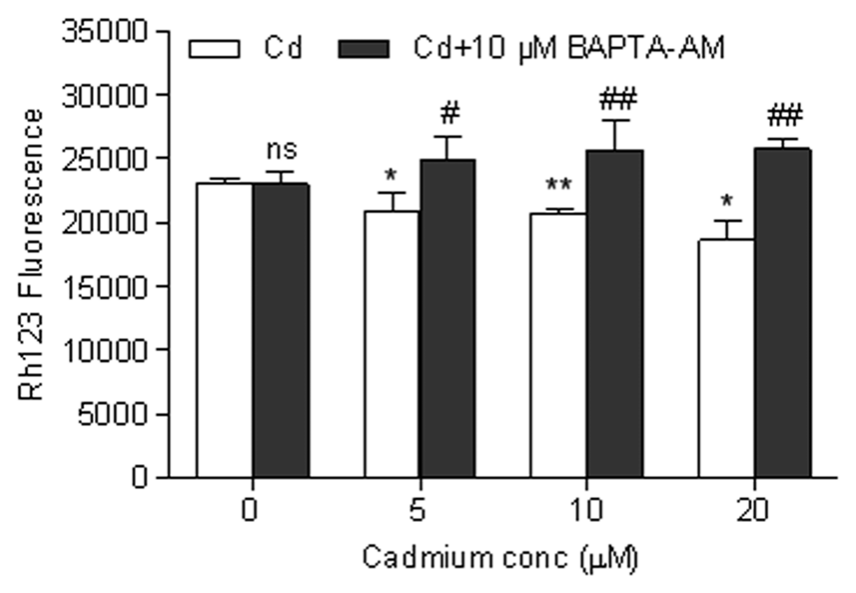
Cd-induced dissipation of Δψm was inhibited by BAPTA-AM. Cells were pretreated with BAPTA-AM (10 µM) for 30 min, followed by treatment with Cd for another 12 h. The Δψm was analyzed by Rh123 staining using flow cytometry. Results were expressed as mean fluorescence. Each bar represents mean ± SD (n = 6). *Statistical significance between control and Cd treatment (5, 10 and 20 µM); ^#^Statistical significance between cells cultured in the absence and presence of BAPTA-AM. *ns* Not significant; *P<0.05, **P<0.01; ^#^ *P*<0.05, ^##^ *P*<0.01 using Student’s t-test.

We examined the effect of Cd on the mitochondrial apoptosis pathway. A decline in Δψm is a morphological characteristic of mitochondrial function impairment. Rh123, a lipophilic cationic fluorescent dye, is selectively taken up by mitochondria and used to assess the mitochondrial ΔΨ of cerebral cortical neurons. As shown in Fig. 6, the mitochondrial ΔΨ decreased in cerebral cortical neurons after exposure to Cd (5, 10 and 20 µM) for 12 h. A significant reduction in mitochondrial ΔΨ was observed (*P*<0.05 or *P*<0.01).

**Figure 7:**
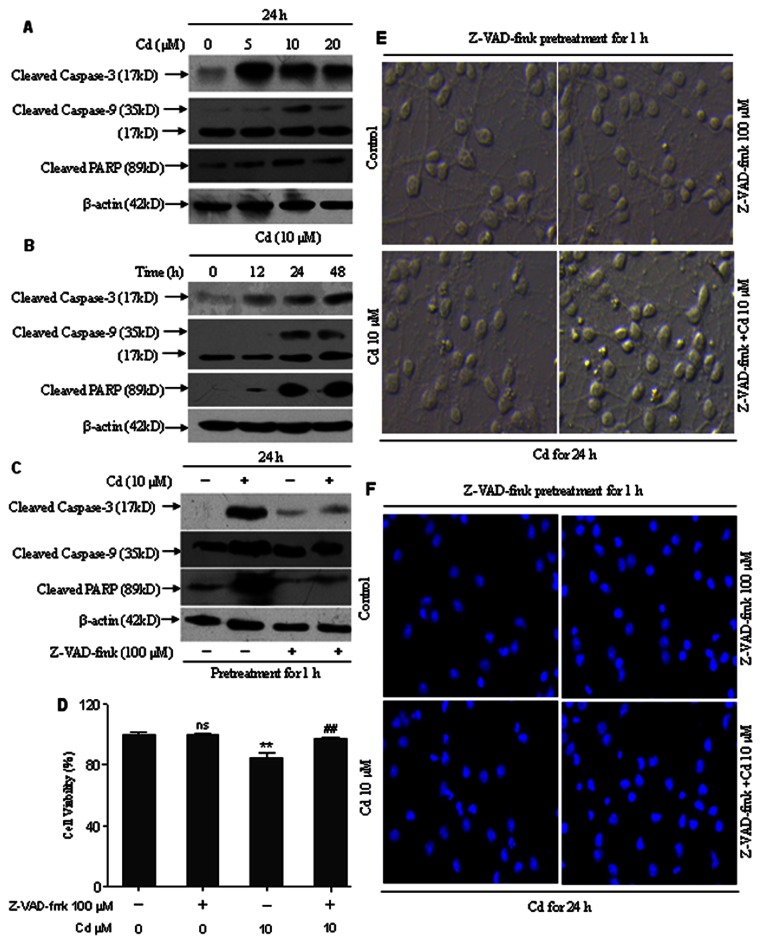
Cd induced neuronal apoptosis by caspase-dependent mechanism. (A and B) Cd increased cleavage of caspase-9, caspase-3 and PARP. Cells were exposed to 0–20 µM Cd for 24 h, or 10 µM Cd for 0–48 h and then analyzed by Western blotting. All experiments were performed twice. (C) A broad caspase inhibitor, Z-VAD-fmk, reverses Cd-induced cleavage of caspase-9, caspase-3 and PARP. Cerebral cortical neurons, treated with 10 µM Cd for 24 h following pretreatment with a pan caspase inhibitor, Z-VAD-fmk (100 µM) for 1 h, were harvested. Cell lysates were analyzed by Western blotting. All experiments were performed twice. (D) Z-VAD-fmk prevented cell death induced by Cd. Cell viability of cerebral cortical neurons treated with/without Cd (10 µM) in the presence or absence of Z-VAD-fmk (100 µM) for 12 h. Results are presented as mean± SD (n = 6). *Statistical significance between control and Cd treatment (5, 10 and 20 µM); ^#^Statistical significance between cells cultured in the absence and presence of Z-VAD-fmk. *ns* Not significant; **P<0.01; *^##^P*<0.01, using Student’s t-test. (E) Z-VAD-fmk prevented morphological alterations induced by Cd. Morphology of cerebral cortical neurons, treated with/without 10 µM Cd in the presence or absence of 100 µM Z-VAD-fmk for 24 h, was assessed using a LEICA inverted phase-contrast microscope (200×) equipped with digital camera. All experiments were performed twice. (F) Cd-induced apoptosis were suppressed by Z-VAD-fmk. Cells were pretreated with Z-VAD-fmk (100 µM) for 1 h, followed by treatment with 10 µM Cd for another 24 h to determine apoptosis by Hoechst 33258 staining under a fluorescence microscope (arrows, apoptotic cells). The original magnification is 200×. All experiments were performed twice.

**Figure 8:**
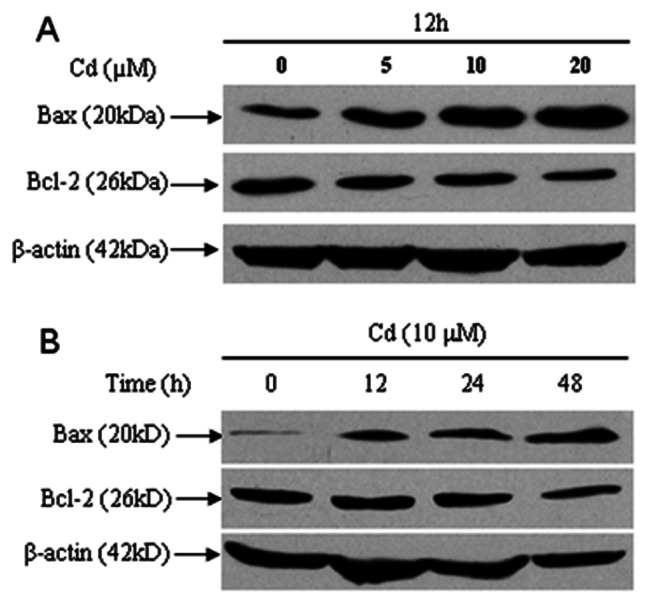
Cd regulated the expression levels of Bcl-2 and Bax. (A and B) Cerebral cortical neurons were treated with Cd (0, 5, 10, 20 µM) for 12 h, or 10 µM Cd for 0–48 h and then assessed by Western blotting. β-actin was used as an internal control. The expression level of Bcl-2 decreased, while that of Bax increased. All experiments were performed twice.

Activation of caspase-9 and -3 and cleavage of PARP are events downstream of the collapse of mitochondria. Our immunoblotting results showed that the levels of activated caspase-9 and -3 and cleaved PARP increased after treatment with 5, 10 and 20 µM Cd for 24 h or 10 µM Cd for 12, 24 and 48 h (Fig. 7A and B). To unveil whether there exists a caspase-dependent mechanism involved in Cd-induced neuronal apoptosis, cerebral cortical neurons were exposed to 10 µM Cd for 24 h after pretreatment with Z-VAD-fmk (100 µM), a pan caspase inhibitor, for 1 h. Cd-induced activation of caspase-9 and -3 and cleavage of PARP were obviously attenuated by Z-VAD-fmk (Fig. 7C). However, when cerebral cortical neurons were pretreated with this inhibitor for 1 h, followed by exposure to 10 µM Cd for 12 or 24 h, MTT assay and morphological analysis revealed that Z-VAD-fmk partially rescued cells from Cd-induced cell death and apoptosis (Fig. 7D–F). In addition, we noticed that Cd increased Bax and decreased Bcl-2 levels in a time- and concentration-dependent manner in cerebral cortical neurons (Fig. 8). Our data suggest that the mitochondrial apoptosis pathway may be involved in Cd-induced apoptosis of cerebral cortical neurons.

**Figure 9:**
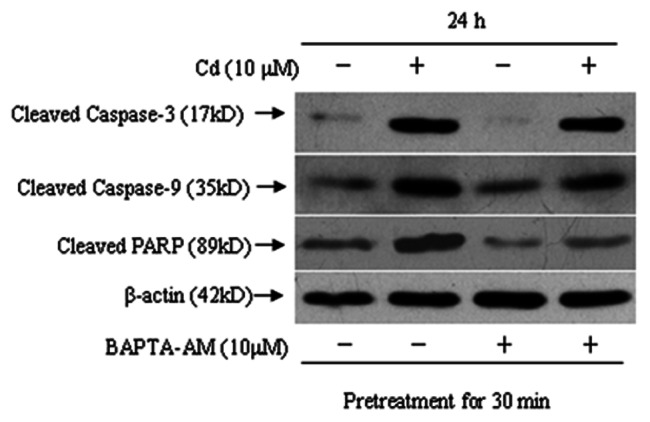
Activation of caspases induced by Cd occurs through calcium signaling. Cells were pretreated with BAPTA-AM (10 µM) for 30 min, followed by treatment with Cd (10 µM) for 24 h. Cell lysates were analyzed by Western blotting. All experiments were performed twice.

An increase in the cytoplasmic level of calcium is known to secondarily alter mitochondrial homeostasis. We found that BAPTA-AM significantly blocked disruption of Δψm in cells exposed to Cd (5, 10 and 20 µM) for 12 h (Fig. 6). Furthermore, cleavage of caspase-9, caspase-3 and PARP were significantly attenuated by BAPTA-AM (Fig. 9), which is in agreement with our observation that BAPTA-AM profoundly prevented Cd-induced apoptosis and cell death of cerebral cortical neurons (Fig. 3). However, increased Bax and decreased Bcl-2 levels were not blocked by BAPTA-AM (data not shown). These data suggest that calcium-mediated mitochondria-caspase b is involved in Cd-induced apoptosis. Moreover, our results collectively suggest that Cd-induced apoptosis of cerebral cortical neurons occurs through a calcium-mitochondria signaling pathway.

## Discussion

Cerebral cortical neurons have been identified as targets of Cd-mediated toxicity [17]. Chen et al [42] have demonstrated that the effects of Cd-elevated [Ca^2+^]_i_ on MAPK and mTOR network as well as apoptosis are through stimulating phosphorylation of calcium/calmodulin-dependent protein kinase II (CaMKII) in primary cortical neurons. In this study, we showed that Cd-induced apoptosis in cerebral cortical neurons is mediated through a calcium signaling pathway. Calcium has been recognized as a ubiquitous intracellular signal responsible for numerous cellular events, such as growth, proliferation, differentiation, and survival/apoptosis [19]. As a second messenger, Ca^2+^ mediates responses of neurons to neurotransmitters and neurotrophic factors, including cell survival or death signals [43]–[45]. Dysfunction of cellular Ca^2+^ homeostasis induces neuronal cell death, and this cellular event has been implicated in many neurodegenerative disorders, such as Parkinson’s disease and Alzheimer’s disease [46]–[48].

Cd has been previously demonstrated to interfere with calcium through different pathways, including inhibition of calcium-dependent ATPases or activation of the inositol triphosphate pathway [13], [22], [23]. Among these pathways, the Na^+^/K^+^-ATPase, and Ca^2+^/Mg^2+^-ATPase play critical roles in intracellular Ca^2+^ homeostasis, in that they pump Ca^2+^ in the cytosol across the plasma membrane, and disruption of this function can result in cytosolic calcium overload [49]. Cd may induce such inhibition of Na^+^/K^+^- and Ca^2+^/Mg^2+^-ATPases activities (Fig. 2B), compromising calcium homeostasis and markedly increasing [Ca^2+^]_i_.

The transition metals frequently cause elevation of [Ca^2+^]_i_ which leads to a cytotoxicity effect [50]. We used BAPTA-AM to chelate intracellular calcium in order to demonstrate that elevation of [Ca^2+^]_i_ plays a crucial role in Cd-induced apoptosis of cerebral cortical neurons (Fig. 3). Xie et al [51] have demonstrated that Cd-induced apoptosis was mediated by the release of Ca^2+^ from intracellular Ca storage. We also observed that pretreatment with 2-APB, a membrane-permeable inhibitor of IP_3_R [52], markedly attenuated Cd-induced [Ca^2+^]_i_ elevation (Fig. 2C), suggesting that Cd-induced [Ca^2+^]_i_ elevation may also involve induction of intracellular release of Ca^2+^ stores. It was reported that Cd could induce an increase of IP_3_, a ligand of IP_3_R, within 5 min after treatment with Cd [53], suggesting that Cd could activate the IP_3_R through increase of intracellular IP_3_ which then triggers the release of calcium from ER. ER is the major calcium storage compartment in the cell. Recent evidence has demonstrated that accumulation of calcium in the mitochondria and apoptosis induced by ER stress can be reduced by pretreatment with an inhibitor of the calcium channel of the ER (IP_3_R) [54], implicating the release of calcium from the ER in promoting apoptosis.

In addition, mitochondria play a role in stress responses and can produce ROS when damaged. Mitochondria are indeed a major source of ROS. ROS production is related to the level of ETC [55]; it is elevated when electron transport is reduced, which occurs in pathological situations [56]. The enzyme COX can serve as an indicator of mitochondrial function. This is because COX dysfunction increases ROS, reduces energy stores and impairs energy metabolism [57]. In addition, COX-I, II, and III levels are quite vulnerable to oxidative damage because their mRNA and protein are in close proximity to the sites of ROS generation [38]. Accordingly, COX-I, II, and III mRNA expression levels were measured using qRT-PCR to investigate the influence of Cd on COX damage. As shown in Fig. 4, the expression levels of COX-I, II, and III in the Cd treatment group were all significantly lower than that in the control group. These results suggest that a decrease in the abundance of specific COX subunits (I/II/III) may be responsible for the COX dysfunction induced by the Cd treatment, which may in turn affect the rates of mitochondrial respiration and ROS production. In metal-induced apoptosis, it is thought that the mitochondria are most pertinent in mediating apoptosis, putatively via metal-induced ROS production [58]. Cd is a well-known inducer of ROS generation in cells [16], [59]. Accumulating evidences indicate that Cd-induced neuronal toxicity is due to induction of ROS, which leads to oxidative stress [4], [36], [37]. Xu et al [17] demonstrated that Cd-elevated [Ca^2+^]_i_ induces ROS, which then triggers apoptosis of neuronal cells. Our experiments also showed that Cd-induced [Ca^2+^]_i_ elevation resulted in the induction of ROS in cerebral cortical neurons after a 12 h treatment with Cd (Fig. 5). BAPTA-AM attenuated Cd-induced ROS (Fig. 5) which suggests a mitochondrial origin for calcium-related ROS production. The elevated [Ca^2+^]_i_ concentration and ROS may cause Ca^2+^ influx into mitochondria which would disrupt normal metabolism of mitochondria, leading to apoptosis and growth arrest.

Intracellular calcium overload may be related to the mitochondrial dysfunction. Mitochondria are vital organelles for cellular metabolism and bioenergetics, but they are also key regulators of cell death [60]. Since mitochondria are the major site of ATP production and mitochondrial ΔΨ is the driving force of ATP synthesis, a breakdown in the mitochondrial ΔΨ could lead to a fall in the ATP levels [61]. The resulting reduction in cellular ATP levels can disrupt ionic homeostasis which can cause an increase in [Ca^2+^]_i_ and subsequent cellular apoptosis/necrosis [62]. Notably, in many (if not all) paradigms of apoptosis, ΔΨm represents the point of no return in the cascade of events that ultimately leads to cell death [63]. In cerebral cortical neurons, we have shown that Cd induces a loss in Δψm (Fig. 6), which precedes the observed apoptosis. In this study, BAPTA-AM was able to prevent this loss in Δψm (Fig. 6) and apoptosis (Fig. 3) from Cd toxicity, suggesting the collapse of mitochondria caused by the elevation of [Ca^2+^]_i_ may play a role in Cd-induced apoptosis.

Members of the B-cell lymphoma (Bcl)-2 family proteins are thought to play regulatory roles in the apoptotic execution of the cells including regulation of the mitochondrial changes during apoptosis [64], [65]. Bcl-2 (an apoptosis-inhibiting gene) and Bcl-2-associated X protein (Bax) (an apoptosis-inducing gene) are two important members of the Bcl-2 family; the ratio between the two determines the survival of cells [66]. There is a negative correlation between the expression of Bcl-2 and Bax. Bcl-2 over-expression leads to cell proliferation, while Bax over-expression leads to cell death [67]. Cd exposure induces significant down-regulation of Bcl-2 expression and up-regulation of Bax [68]. In this study, we showed that Cd increased Bax and decreased Bcl-2 levels in a time- and concentration-dependent manner in cerebral cortical neurons (Fig. 8). These cellular events were not blocked by pre-treating cerebral cortical neurons with BAPTA-AM (data not shown). A number of studies have indicated that Bcl-2 prevents cytosolic calcium increase, while Bax may promote this increase during apoptosis [69], [70]. Cd reduces the expression of Bcl-2 and increases the expression of Bax, which results in an overload of Ca^2+^ in the mitochondria and promotes the opening of permeability transition pores. Mitochondria will then swell, with their outer membranes collapsing and exiting into the cytoplasm, which would consequently trigger apoptosis. In conclusion, Cd-induced apoptosis and [Ca^2+^]_i_ elevation of cerebral cortical neurons may involve regulation of the expression of Bcl-2 family proteins.

Caspases, a family of cysteine-dependent aspartate-directed proteases, play critical roles in the initiation and execution of apoptosis [71]. Among various caspases, caspase-3 appears to be the main effector, due to (i) its critical role in inducing characteristic apoptotic changes, including chromatin condensation, DNA fragmentation, and formation of apoptotic bodies, and (ii) its close association with many other mediators in apoptosis such as caspase-9, cytochrome c, and PARP [72]. [Ca^2+^]_i_ elevation may have mediated Cd-induced apoptosis through [Ca^2+^]_i_-ROS-JNK-caspase-3 and Ca^2+^-mitochondria-caspase signaling pathways [13], [73]. Recent studies have shown that Cd induces caspase-dependent and -independent apoptosis of PC12 and SH-SY5Y neuronal cells [36]. Furthermore, the caspase-3 pathway is involved in Cd-induced apoptosis in cortical neurons [74]. Our results demonstrated that Cd-induced apoptosis in cerebral cortical neurons involves the activation of caspase-3, caspase-9 and the cleavage of PARP. Pretreatment of the cells with a broad-spectrum caspase inhibitor, Z-VAD-fmk, did prevent cleavage of caspase-3, caspase-9 and PARP (Fig. 7A–C), but partially prevented Cd-induced cell death and apoptosis (Fig. 7D–F), revealing that Cd induces neuronal apoptosis through caspase-dependent mechanism. Similar findings have been reported in other neuronal cells, such as PC12 cells [36] and oligodendrocytes [29]. BAPTA-AM was able to reduce caspase activation and the cleavage of PARP (Fig. 9), suggesting apoptosis in Cd-treated cerebral cortical neurons is mediated by calcium-mitochondria-caspase signaling.

In the present study, we observed that Cd-induced apoptosis is associated with calcium-induced massive production of ROS, dissipation of ΔΨm, cleavage of caspase-9, caspase-3 and PARP. Collectively, our results demonstrate that Cd-induced apoptosis is mediated by calcium signaling pathway and calcium-mediated apoptosis occurs through the mitochondria-caspase signaling pathway. In conclusion, our results provide a molecular evidence to demonstrate a novel finding that Cd induces calcium-mitochondria-caspase-mediated apoptosis.
